# Rethinking district-level governance of malaria in Ghana: A narrative review

**DOI:** 10.1371/journal.pgph.0000764

**Published:** 2022-07-13

**Authors:** Nii Ayite Coleman

**Affiliations:** Consultant, Accra, Ghana; University of Birmingham, UNITED KINGDOM

## Abstract

The goal of global malaria programming is to eliminate and eventually eradicate the disease. Achieving this global goal requires eliminating malaria in individual endemic countries. This paper, based on the observations of current and former implementers of malaria programs at district level, examines Ghana’s malaria strategy to determine its adequacy for the elimination of malaria in the country, with a focus on the governance of district health systems. The paper argues that Ghana’s malaria strategy is medically oriented, focusing predominantly on diagnosis and treatment of the disease. The strategy ignores determinants of malaria that are related to lifestyle and environment. Furthermore, there is limited engagement with other district-level stakeholders, and what engagement does occur is neither systematic nor sustained. Ghana’s malaria strategy therefore requires a systematic rethinking to mobilize the participation of communities in district governance for malaria. The paper proposes several possible actions to restructure district governance of malaria. In Ghana, the malaria program should engage with key stakeholders in districts, using a systematic and sustained approach, to strengthen multisectoral action and community participation. This will require new accountability relationships for malaria progress within communities and among District Assemblies, district health authorities, and communities. Malaria programs in other African countries may also need to be similarly redirected towards community health governance for malaria progress. Simultaneously, global health and malaria agencies should redefine malaria as a social problem and collectively adopt a social determinants approach to strengthening national malaria programs. Pursuing the goals of elimination and eventual eradication of malaria without incorporating effective control of mosquito breeding and public health regulation is a fundamentally flawed approach. Progress on malaria requires a paradigm shift, from a medical perspective to a social determinant-informed approach with sustained and systematic engagement of all stakeholders in local communities.

## Introduction

Primary health care (PHC) is the cornerstone of health development in Ghana and the key mechanism for attaining universal health care coverage in the country. Among its many components, PHC includes essential health services, health education, immunizations, prevention and control of locally endemic diseases, and the provision of essential drugs. Furthermore, it is supposed to be “made universally accessible with the full participation of the community” [1]. The WHO has emphasized this point:

Measures have to be taken to ensure free and enlightened community participation, so that notwithstanding the overall responsibility of governments for the health of their people, individuals, families, and communities assume greater responsibility for their own health and welfare, including self-care…. Governments, institutions, members of health professions as well as all agencies involved in health and development, will therefore have to take measures to enlighten the public in health matters so as to ensure that people can participate individually and collectively, as part of their right and duty, in the planning, implementation and control of activities for their health and related social development

[2].

The concept of participation has been further elaborated into a focus on community health governance. As national health systems and global health programs strive to improve the performance of their interventions, health systems governance is receiving increasing attention. The proliferation of work on health systems governance over the past decade is based on the expectation that good governance ultimately leads to better health outcomes [3]. Building on the PHC “village health committee” model, community health governance is understood to include all stakeholder groups in the community and encompasses accountability. Community health governance nurtures accountability relationships between the entire spectrum of stakeholders in the community and thereby engenders better district health systems governance.

The concept of governance is universal in communal contexts such as families, clans, villages, associations, companies and nation-states. In each communal context, representatives are chosen to act on behalf of the collective [4]. The domain of governance can be considered the relationships between the representatives and the represented aimed at ascertaining responsive and effective action in the interests of the collective. There are multiple detailed definitions of governance. Lehman and Gilson’s definition emphasizes the multidimensional nature of governance, stating that governance entails: “ensuring that strategic policy frameworks exist and are combined with effective oversight, coalition building, regulation, attention to system-design and accountability” [4]. In practice, governance of a country is dependent on the political arrangements at national level, often enshrined in a national constitution, that define the relationship between the government and the governed. In Ghana, constitutional arrangements for national governance form the basis for governance in all sectors of the economy, including health [5]. It also defines arrangements for sub-national levels, including the district level [6]. Governance is also recognized as a key element that facilitates the functioning of the health care sector. In health, as in many other sectors, governance is not just a top-down function but needs to be operationalized by individuals at all levels in the health system. In this case, the importance of governance in district (or similar level) health systems is increasingly being recognized as crucial to the achievement of universal health care coverage as well as sustained improvements in the performance of health interventions and outcomes, including malaria [7].

This paper explores the challenges and opportunities inherent in the district-level governance of malaria interventions in Ghana. First, it presents an overview of Ghana’s malaria strategy and assesses the nature and scope of the malaria program’s engagement with district- and lower-level communities, authorities, and other stakeholders. Second, it considers the relationships between and within stakeholder groups in the district. Then it discusses the challenges of district health system governance to the implementation of the malaria program. The paper concludes by making suggestions to facilitate improvement in the governance of the district health system in Ghana and comments on the implications of this situation for other malaria-endemic countries.

## Methods

The paper was prepared for the governance working group of the “Rethinking Malaria in the Context of COVID-19” process convened by Harvard University (see: https://www.defeatingmalaria.harvard.edu/rethinking-malaria/). I was tasked by the working group to draw on my 34-year career serving in the Ministry of Health in Ghana at district, regional, and national levels to provide observations on district-level governance of malaria programming. I have worked as a medical provider at district hospitals, a District Medical Officer of Health, a Regional Director of Health Services, and as Director of Policy, Planning, Monitoring and Evaluation of the Ministry of Health. I also served as the Director of the Accra Metropolitan Public Health Department. In preparing the paper, I solicited the viewpoints of malaria program implementers by interviewing four individuals who all worked in one district. The interviewees were: a regional coordinator for the malaria program, a District Assembly malaria focal point person, a District Assembly public health official, and the medical superintendent of a hospital in the district. The interviewees included two men and two women; of these, one was early career, two were mid-career and one was retired. During the semi-structured interviews, I asked each participant to reflect on the malaria situation in the district, the roles of their institutions and other stakeholders in combating malaria, the key challenges faced, and their ideas for addressing the identified challenges. I reflected on these comments and integrated their views with my observations. (The interviews are cited as “personal communication” in the paper to maintain the interviewees’ anonymity.) A literature search was conducted in two databases (PubMed and Web of Science) using the search terms “Ghana”, “district”, “governance” and “malaria”. However, no directly relevant papers were found. All published documents used as sources in preparing this paper are cited. The findings and commentaries presented herein are based on triangulating policy documents and the observations and experiences of malaria program implementers.

### Ghana’s malaria strategy

Ghana is classified as being in the malaria “control” phase. Malaria-specific mortality among children less than 5 years old has declined from 14.4% in 2000 to 0.6% in 2012. The same level of success, however, has not been achieved with malaria morbidity [8].

A review of Ghana’s national malaria strategy was conducted in 2013. Based on recommendations from the review report [9], as well as various new interventions emerging at the global level, Ghana’s National Malaria Control Program developed the National Malaria Control Strategic Plan for 2015–2020, which was published in August 2014. [9] The plan’s overall aim was to reduce the country’s malaria morbidity and mortality burdens by 75% by the year 2020 (using 2012 as baseline). Specific objectives included [10]:

1. To protect at least 80% of the population at risk with effective malaria prevention interventions by 2020.
2. To provide correct diagnosis to all suspected malaria cases and prompt and effective treatment to 100% of confirmed malaria cases in accordance with treatment guidelines by 2020.
3. To strengthen and maintain the capacity for program management, partnership, and coordination to achieve malaria programmatic objectives at all levels of the health care system by 2020.
4. To strengthen the systems for surveillance and monitoring and evaluation in order to ensure timely availability of quality, consistent and relevant malaria data at all levels by 2020.
5. To increase awareness and knowledge of the entire population on malaria prevention and control so as to improve uptake and correct use of all interventions by 2020.

In general, the strategic plan sought to consolidate gains, accelerate malaria control in high transmission areas, and move towards establishing lower-transmission areas in Ghana by the end of 2020 [11]. The strategy and the program overall are both dominated by a medical approach–that is, malaria is viewed as a disease requiring treatment in a health care facility.

Using a medically-oriented approach to malaria is, in my opinion, a serious mistake that will result in keeping Ghana in the malaria control phase. It is an inadequate strategy if the goal is to achieve elimination and eradication of malaria. Malaria is more than a medical problem; it is also a social problem directly influenced by social determinants including lifestyle and environmental factors [12]. Indeed, malaria is as much an outcome of environmental and lifestyle factors that result in poor sanitation and lack of infrastructure as it is of insufficient knowledge or lack of access to health care. One result of focusing only on malaria as a health care problem is uncontrolled mosquito breeding that leads to extensive human exposure to the vector in communities. Malaria therefore cannot be eliminated without eliminating major mosquito breeding sites. To pursue malaria elimination in Ghana necessitates addressing mosquito breeding in every community. Setting a goal of elimination, and eventually of eradication, of malaria without including effective control of mosquito breeding and public health regulation as a key component of the strategy is fundamentally flawed. Elimination of malaria requires a paradigm shift from a medical perspective to a social determinant approach. A social determinant approach to malaria would engender sustained and systematic engagement with the full spectrum of stakeholders in every community.

Although Ghana’s national malaria strategy states an intention to increase the population’s awareness and knowledge about malaria prevention and control [9], in practice the malaria program’s engagement with stakeholders has been constrained by its overall orientation towards medical interventions. For example, the Social and Behavior Change Communication (SBCC) Strategy for the National Malaria Control Programme has a limited scope of activities focused mostly on national and regional mass media campaigns [13]. Interpersonal communication activities conducted at facilities and in communities focus on adherence to national case management guidelines and prompt health care-seeking. School-based interpersonal communication activities, conducted in tandem with distribution of Insecticide-Treated Nets (ITNs), focus mostly on correct and consistent use of ITNs and ITN care practices. The communication strategy included focusing on “advocating to political leaders, policy makers, opinion leaders and corporate bodies for support for malaria control” and “sustaining communication, education, and community mobilization to increase knowledge among the general population to enhance uptake of malaria prevention interventions” [13]. However, these activities rarely, if ever, address community-based social and environmental strategies for malaria elimination. Ghana should be supporting districts to design and implement these strategies at the district level.

### District-level engagement in malaria interventions

Ghana, a parliamentary democracy, has 16 administrative regions that are further sub-divided into 216 districts. Each of these is governed by a District Assembly. Community mobilization on health is predominantly implemented through the District Health Service, and engagement with stakeholders (such as health care providers, community organizations, local leaders, the private sector and others) should be carried out by the District Health Management Team (DHMT). The Ghana Health Service and Teaching Hospitals Act (Act 525) of 1996 also made provision for a certain degree of engagement with some stakeholder groups through the creation of a District Health Committee [6]. Section 23 of the Act establishes a District Health Committee with representation from the District Health Service, the District Assembly, local religious and traditional leadership, and health care workers from the public and private sector. It also specifies that at least one of the minimum two community representatives is a woman. The core function of this District Health Committee is essentially advisory. Section 24 (1) of the Act says: “A District Health Committee shall advise the District Director of Health Service in the performance of his functions in the district and shall perform such functions of the [Ghana Health Service] Council in the district as the Council may assign to it.” [6] According to interviewees and my experience, however, District Health Committees are not in place in all districts. As a result, district-level engagement with potential stakeholders is widely variable and often ad hoc.

Concerns about district-level engagement in health overall are amplified in the case of malaria. The national malaria program appears to have limited engagement with district health systems; furthermore, it has limited stakeholder participation in its programming. This results in the malaria program appearing to district-level stakeholders to be prescriptive and lacking accountability to its intended beneficiaries. A few examples: First, the malaria program is not perceived to be truly rooted in the communities. It is associated only with the implementation of certain specific activities, such as distribution of ITNs or larviciding. One person working as a “District Malaria Focal Person” recently acknowledged this, stating that they only engage stakeholders for specific activities. Instead of regular and sustained interactions with facilities, for example, the focus person noted that they only “deal with the hospital when there are any problems with data.” (Personal communication, 2021) Second, in some districts, focal point persons arrange for larviciding to be carried out by private companies without the full knowledge and participation of the Public Health Department of the District Assembly. (Personal communication, 2021)

This lack of effective engagement of stakeholders in the malaria program is partly related to the national strategy’s emphasis on medical solutions to malaria morbidity and mortality. It is also partly due to the rigidity of the program’s protocols. However, the context in each district is also important. In particular, the continued absence of District Health Committees (despite having been mandated in 1996) demonstrates a lack of institutional capacity that prevents the Ghana Health Service in general, and the national malaria program in particular, from truly engaging with a broad spectrum of stakeholders over the long term.

Concerns about national capacity and overly medicalized approaches to health problems are not new problems. In September 1976, Professor Comlan Quenum, addressing the WHO Regional Committee for Africa, said:

We can no longer consider health programs without reference to other sectors of socioeconomic development…. The myths of the past imposed a dichotomy between politics and health, a dichotomy between socioeconomic development plan and the health program, as if health, which is essentially a social sector, could be dissociated from the national will expressed through a particular political choice.…We must also devise new procedures for strengthening health services. This requires a special effort to make the most of local resources, particular manpower. It is fair to say that there can be no development without using all human resources to full advantage, i.e. without material and cultural development of the people as a whole. Regrettably, the existing health delivery systems exclude the communities concerned; their health and their environment can be improved only if they play an active part in the systems organized for that purpose. That is why all our future efforts must be aimed at enlisting authentic community participation so as to help its members become aware of their needs and to encourage them to cooperate in finding solutions and managing services.

[1]

These words still ring true over 40 years later with respect to malaria. The elimination and eventual eradication of the disease require a more nuanced appreciation of malaria as a social problem and a call for a coherent community response. Although Ghana has yet to fully realize this, it does have many of the necessary structures in place. Now we need to recognize and organize them to make full use of all available resources in reducing the toll of malaria.

### Relationships between and within stakeholder groups in the district

Various malaria stakeholder groups exist at the community and district levels in Ghana. Several types of stakeholder groups are already active in the communities, including households, youth organizations, women’s groups, religious organizations, elected local officials, and traditional leaders. Other key stakeholders are health care service providers, district health authorities, and the district assembly. This section describes how each of these stakeholder groups can and should be engaged in anti-malaria efforts.

### Households and community-based organizations

Malaria is endemic in Ghana and every person living in the country is at risk of contracting it. Thus everyone in Ghana has an interest in the control, elimination, and eventual eradication of malaria. It is the number one killer of children and the leading cause of reported morbidity in the country. Households and local associations, such as women’s groups, youth organizations, and religious bodies, are frontline stakeholders in malaria in cities, towns and villages across the country.

Individuals and groups also often have other stakes in malaria besides self-interest. Civil society organizations, both indigenous and international, are growing in numbers and have emerged as an important stakeholder group representing the people whose voices are not being heard. For example, there is a National Coalition of NGOs in Health and a specific coalition of NGOs in malaria. Increasingly, civil society organizations are becoming active in the implementation of social programs, including to address malaria, in districts across the country. These efforts require support from the national program as well as from donors.

### Health care service providers

Health care services in the district are provided at facilities with varying capabilities and owned by various groups including government, private and religious organizations. Public health care services are provided by the District Health Service (DHS), usually through a network of health centers, clinics, Community-based Health Planning and Services (CHPS) compounds and outreach centers, with a district hospital serving as a referral facility. This public network provides allopathic medical care and preventive medicine including maternal and child health services and immunization.

Allopathic medicine does not have a monopoly over the diagnosis and treatment of malaria. Indeed, a few district hospitals around the country are now also practicing herbal medicine. National legislation on traditional and alternative medicine has supported pluralism in health care delivery. As a result, although allopathic medicine is dominant, other forms of alternative medicine and health care practices are also increasingly available [14]. The production and use of traditional herbal preparations are growing, and traditional medical practitioners (such as Traditional Birth Attendants, herbalists, bone-setters, and spiritualists) are well patronized. All health practitioners, whether in allopathic or other traditions, should be fully supported to help their patients and communities to understand, prevent and treat malaria effectively.

### District health authorities

The district health system is characterized by multiple care systems, varied ownership, and fragmented leadership. There is no single district health authority. Instead, the current legislation regime for districts mandates both a District Health Service (of the Ghana Health Service) and a Public Health Department (of the District Assembly). The health service’s District Health Management Team (DHMT) oversees health centers, clinics, CHPS compounds and outreach services, and the district hospital. The district hospital, under a medical superintendent, provide a range of basic health care services, including emergency surgery, blood transfusion and laboratory services. The Public Health Department (PHD) of the District Assembly, made up of health inspectors, is responsible for public health services, oversees sanitation and waste management, and enforces public health regulations [6]. In practice, district-level health leadership and governance is fractured into three core services: basic medical care and preventive medicine (under the DHMT), district hospital care under medical superintendent, and public health services under the PHD. Each has a different source of funding and interactions among them may be ad hoc. Yet malaria requires interventions by all three areas of health service, plus others in other sectors.

### Traditional council

Traditional leaders—chiefs and their councilors—based in villages and communities are the frontline authorities. For the majority of Ghanaians, traditional leaders are regarded as representatives of the people in their respective towns and villages. Ghana’s traditional system of government predates colonialism [15]. For example, the Asante ethnic group has long had a highly organized system of government in place:

The Asantes were politically united under the Asantehene before colonial rule…At the side of the Asantehene stands the Asanteman Council, composed of paramount chiefs of the member states of the Asante confederacy. The paramount chiefs assist the Asantehene in his direction of the affairs of the Asante nation. The paramount chiefs also hold positions in their own states. As paramount chiefs of their states, they govern their people with a council comprised of elected representatives of the state. Similarly, sub-chiefs and village chiefs serve their smaller communities with the help of elected representatives from the local communities. Within these communities the town chief or village head serves the people as the leader of the community. But he consults with a council which is made up of the heads of the respective lineages who are resident in the village or the community. In other words the political structure of the Asante social system radiates the authority of the Asantehene through to the level of the extended family network.

[16].

A version of this political system “remains in evidence today” in that the central government relies on chiefs to deal “with traditional matters” [16]. Traditional political authority is enshrined in Ghana’s constitution (Article 270 of the 1992 Ghana constitution) [5] and has been institutionalized in the Ministry of Chieftaincy Affairs. The Traditional Councils have strong stakes in the well-being and development of their people. The endorsement of chiefs, though informal, is important for the successful implementation of any public sector project or program in local communities. Strong support from traditional leadership is therefore critical to any effort to address malaria.

### District assembly

Ghana’s current efforts at decentralization began in 1988 with the promulgation of PNDC Law 207. In 1992, Article 240 of the Ghana constitution further stipulated “a system of local government and administration which shall, as far as practicable, be decentralized” [5]. The Local Government Act of 1993 (Act 462) then sanctioned a body called the District Assembly (DA) to be responsible for overall development in the districts through the exercise of deliberative, legislative and executive powers [17]. The DA membership comprises two-thirds elected and one-third appointed members, and is headed by a District Chief Executive (DCE) appointed by the national President [5]. As one scholar noted, “The District Assemblies were to be the foundation on which Ghana’s new democracy was to be erected” [18].

The DA has two committees, the Audit and the Executive Committees. The Executive Committee (EC), which is headed by the DCE, serves as the cabinet. The EC has five statutory sub-committees: Finance and Administration, Development Planning, Social Service, Justice and Security, and Works. The role of the DA is to coordinate and oversee implementation of public programs by decentralized departments of the Public Service. The departments provide technical guidance and carry out the actual implementation of policies, projects and programs as mandated by the DA and the national Government. The DA also has locally-elected sub-district councils.

Coordination and oversight of social services is carried out by the Social Services Sub-Committee (SSSC) of the District Assembly. It comprises heads of district departments and agencies, including health, youth and sports, education, water, community development, physical planning, agriculture, disaster prevention and management, and social welfare. Beyond coordination, the SSSC also has a strategic function. It is expected to examine and collect data about the full range of social welfare concerns in the district, and to propose short-, medium- and long-term social development plans for the district for consideration by the DA. Clearly, malaria concerns should be a major focus for the SSSC.

The DA system overall has faced numerous challenges, of which “fiscal decentralization remains one of the most intractable problems” [18]. In essence, the absence of fiscal decentralization is a major roadblock in the evolution of decentralized governance in Ghana. The DA is funded through an irregular and unreliable District Assembly Common Fund and the meager local taxes it is able to collect. The DAs have severe budgetary constraints and thus lack effective control over departments and programs in their districts. Without meaningful fiscal decentralization, the decentralized departments of the DA are funded through their respective sector Ministries. As a result, district heads of departments and agencies have stronger “vertical” alliances—that is, to higher levels of their sectoral ministries—than they do to the District Assembly.

In practice, the absence of fiscal decentralization has paralyzed the DA, rendered the SSSC weak and ineffective, and made the DA and its sub-district structures (called zonal councils and unit committees) almost redundant in their communities. One particular result of the ineffectiveness of the SSSC is that locally-coordinated multisectoral action is incoherent. There is an absence of accountability relationships between and within the District Assembly, the district health authorities, and the communities.

The DA’s inability to effectively coordinate among decentralized departments poses a significant challenge to the malaria program (and other programs dependent on multisectoral collaboration). The fractured district health leadership and the complicated financing architecture of the district turn departmental programs into vertical programs, hampering the development of alliances and coalitions, and forestalling multi-sectoral collaboration.

### Challenges of district health systems governance

Ghana’s administrative decentralization process has a long history. It began with efforts by the colonial administration to establish a local governance system. After an initial period of “Indirect Rule” through the Traditional Councils, disagreements over taxation and other issues between the colonial administration and the Traditional Councils led to the establishment of an alternative and parallel modern local government system; the 1944 Native Authority Ordinance neglected the traditional authorities and put the colonial administration in direct control of the localities [15].

Since Ghana achieved independence in 1957, its subsequent governments have focused on developing a local government system that is an appendage of the central government. Traditional political authority, though legitimate, has been marginalized in the national development agenda. As a result, traditional political authority and modern local government offer disparate political leadership at the community level—this impedes systematic local development. The existence of parallel traditional and modern political authorities presents formidable challenges that are evident in the complex relationships among three key sets of stakeholders: the DA, district health authorities, and communities.

Three key challenges emerge during interactions among the stakeholders.

1. **Fractured political leadership and unaccountable frontline workers**
  The challenges of governing district health systems are inseparable from the burdensome challenges of local government reforms and decentralization in Ghana and the perennial journey “towards democratic local government structures, and accountable systems of public administration that are able to deliver on the developmental demands of the people” [18]. Traditional and modern political leadership both have constitutional legitimacy—and both have often failed to provide meaningful collective community leadership. The decentralization policy has left a gap in local governance by failing to create a working interface between traditional and modern political authorities at the community level. This, in turn, leaves frontline workers without clear areas of authority and lines of accountability. Malaria programs may not be able to function effectively without clear leadership and access to resources.
2. **Weak DAs and ineffective SSSCs hamper intersectoral collaboration, coordination and efficiency**
  The DAs do not have strategic policy frameworks that foster multi-sectoral collaboration and coordination of the implementation of departmental programs. In lieu of an overall district development agenda, disparate projects and programs of the central government are implemented in isolation by various ministries, departments and agencies. The notion of a composite district budget, controlled by the DA and designed to meet the specific priorities and context of a given district, remains an idea that would require fiscal decentralization to become reality. In the absence of a district agenda in every district, there cannot be either coherent district health agendas or multisectoral district malaria agendas.
3. **Legal regime fractures district health leadership**
  As noted, three components of the health system—medical care, preventive medicine and public health services—operate independently in districts without effective arrangements to foster sustained collaboration and coordination of health programs in the district. There is no joint planning for health in the district, and there is no district health strategy. As a result, the district health system does not “own” the malaria program, nor does it have the capacity to engage the full range of multisectoral stakeholders.

### The way forward—For Ghana and in other endemic regions

Exploring the challenges of district health systems governance for malaria raises many bigger questions—about the relationship between central and district governance, about the political economy of global health, about local development and the delivery of social services, about health as a catalyst for community development, about the relationship between traditional political authority and modern political authority, and about the governing of community health. Such questions may seem intractable. However, devising strategies for effective community responses to malaria helps focus in on key issues.

In Ghana, it is clear that an effective approach to malaria control, and eventually to malaria elimination, requires establishing improved relationships between traditional and modern political authorities at the district level in Ghana. The constraints of stakeholder engagement encountered by the malaria program is an indication of the need for better governance of malaria in Ghana; the need for “the alignment of multiple actors and interests to promote collective action towards an agreed upon goal” of the malaria program [19]. Malaria control in Ghana has been managed by experts in the health sector. For the elimination and eradication of malaria, better governance of community health is essential. Bringing the various parties together to focus on malaria could both have a positive impact on reducing malaria morbidity and mortality further and could provide opportunities to delve into those other big questions.

In 2000, Ahwoi identified the need to promote popular participation by shifting processes of governance towards consultation, noting that:

The trends in Local Government Reforms and Decentralisation in Ghana today are quite clear. They are towards democratic local government structures and accountable systems of public administration that are able to deliver on the developmental demands of the people. There have been very positive achievements, but a lot also remains to be done. What we all ought to remember, however, is that decentralisation is a process, not an event. We must therefore not throw up our hands in despair when we confront obstacles. Ours is to devise strategies to overcome those obstacles

[18].

Brinkerhoff and Bossert [20] identified four principles that could assist in changing the culture of governance of health systems. First, governance rules should ensure some level of accountability of the key actors in the system to the beneficiaries and the broader public. Second, health governance involves a policy process that enables the interplay of the key competing interest groups to influence policy making on a level playing field. Third, health governance requires sufficient state capacity, power, and legitimacy to manage the policy making process effectively. Finally, governance depends upon the engagement and efforts of non-state actors in the policy arena as well as in service delivery partnerships and in oversight and accountability.

### Community health governance

A 2009 study noted that “Good governance in health requires the existence of standards, information on performance, incentives for good performance, and, arguably most importantly, accountability” [21]. Ackerman described accountability as “a proactive process by which public officials inform about and justify their plans of action, their behavior and results, and are sanctioned accordingly” [22]. District health systems governance should create accountability relationships between and within communities, district health authorities, and district political authorities. Effective governance of district health systems depends on better governance of community health in towns and villages in the district. Community health governance requires establishing accountability relationships between and within traditional leaders, elected local officials, civil society organizations, community-based organizations, and health care service providers. Community health governance offers the pathway to engaging all stakeholders in a systematic, sustained and dynamic manner.

For malaria, community health governance entails convening stakeholders in the community for several intersecting purposes: to determine what to do about mosquito breeding; to oversee activities to control mosquito breeding; to monitor progress in the control of mosquito breeding; to ensure the community has access to the diagnosis and treatment of malaria; to monitor the number of malaria cases and deaths from malaria; and to hold the malaria program staff and public officials (i.e., the District Assembly and the District Health Service) accountable. Community health governance would strengthen the mobilization and effective use of human and financial resources within the community and engender public-community partnership for health development, including malaria.

This notion of community health governance is clearly within the context of the national decentralization and development frameworks articulated in the Local Government Act of 1993 [17] and related subsidiary legislation, which aim to move “towards democratic local government structures, and accountable systems of public administration that are able to deliver on the developmental demands of the people” [18].

Ultimately, I argue for nurturing community health governance as an integrated component of the national malaria program. This supports a vision of communities with unified political leadership and established accountability relationships between community political leaders, civil society, and frontline service providers. Building these structures for malaria will benefit both malaria and other community health endeavors. To achieve the vision of community health governance, three key strategies are suggested, together with illustrative activities:

1. **Foster alliances and coalitions to govern community health**
  - Facilitate alliances between elected local officials (assembly, zonal council and unit committee members) and traditional leaders (chiefs and elders) in the communities to support and coordinate community malaria projects and programs, to oversee frontline malaria workers in the community, and to ensure efficient use of available resources
  - Foster coalitions between community groups, religious groups, and other civil society groups
  - Nurture community health governance by establishing accountability relationships between traditional and elected leaders, civil society, and frontline service providers
2. **Develop the capability of the Social Services Sub-Committee of the DA to engender multisectoral action**
  - Strengthen the SSSC’s capability to coordinate and oversee the implementation of malaria programs in the district through strategic and sustained technical support, continuing education, and logistical support
  - Facilitate the development of a strategic policy framework for malaria and health
3. **Promote more unified district health leadership under a District Medical Officer of Health (DMOH) to develop a district strategy**
  - Foster more unified leadership of the district health system through joint planning, monitoring of implementation and assessment of performance in malaria programs
  - Recruit and develop DMOH for district health leadership
  - Promote the development of district health and malaria strategies by bringing diverse components of the malaria program into a single district malaria implementation strategy with oversight from the SSSC of the DA

Given adequate institutional incentives, effective engagement can strengthen direct accountability relationships among communities, DAs and district health authorities [23].

In the medium term, improvement in some dimensions of malaria governance (such as coalition building, oversight and accountability) has the potential to enhance program implementation and result in better health outcomes. Effective community health governance has the capability to enhance the implementation of Ghana’s malaria program, to improve the chances of malaria elimination and eradication, and to engender community development.

## Conclusion

District- and community-level health governance is the next logical step in the development of health systems in Ghana. The concept builds on the foundations of PHC policies and programs, community participation and village health committees, alongside the more recent development of district health systems. Community health governance is the mechanism to realize a true paradigm shift, from a medical orientation to a social determinants approach. Malaria would make an ideal test case for this. A paradigm shift to social determinants approach would enable a reorientation of how malaria is handled at the community, district, national and global levels.

This raises broader implications for consideration in Ghana, as well as in other African countries, and within the global malaria agencies. First, the Ghana malaria program must engage systematically and in sustained ways with key stakeholders in the districts in order to make progress towards elimination and eradication of the disease. This would require nurturing, fostering and facilitating alliances and coalitions among stakeholders and strengthening multisectoral action. The malaria program must invest in establishing accountability relationships within the communities and between the District Assembly, the district health authorities and the communities.

The analysis in this paper has some limitations due to its reliance on observations from a small number of interviewees in a single country and my personal experiences as a public health official during the past three decades. However, malaria programs in other African countries with similar governance systems may be able to learn from Ghana if it redirects towards community health governance. Similar district health systems exist in most former British colonies and decentralization is taking place in most of those countries. As a result, the specific country contexts for malaria programs are similar to what pertains in Ghana, making these proposals relevant and applicable.

Finally, improving malaria governance at the district level in African countries has implications for the governance of malaria at the global level. This analysis supports the necessity of rethinking “the malaria problem” by global malaria agencies. The continued high malaria burden is a fundamental problem for African societies. Malaria is a social problem, and an indicator of social underdevelopment, poor living standards, and unacceptable quality of life.

The urgent responses to the global COVID-19 pandemic should open the global policy window to enable consideration of fresh policy initiatives to more forcefully address the longstanding problem of malaria in Africa. Global malaria agencies must, as a matter of urgency and with unity of purpose, redefine malaria as a social problem and collectively adopt a social determinants approach to the development of national malaria programs.

Beyond opening the global policy window, COVID-19 also offers valuable lessons about strengthening local health systems and facilitating community organization in preparedness for the next pandemic. Redefining the malaria problem as a social one would expand the options for addressing the malaria problem beyond health care delivery to include community response. Strengthening district-level governance for malaria, however, will require additional research and understanding on a number of topics, as suggested in Box 1, to help improve the evidence base on how to address malaria governance challenges in Ghana and beyond. With adequate institutional incentives, a community response initiative would facilitate the forging of relevant alliances and coalitions, engender the alignment of multiple stakeholders and interests to promote collective action, and lay the foundation for establishing community health governance. We may find a post-COVID-19 policy window that provides an opportunity to put community health governance on the global malaria policy agenda.

**BOX 1.**

**Topics for research on district-level malaria governance:**

- Assess the coherence of national and district malaria elimination and control policies
- Assess the roles of community health committees in local malaria elimination efforts
- Identify effective approaches to strengthen accountability of malaria program to district stakeholders, including communities
- Define roles and relationships for the District Assembly, as a local democratic institution, and national malaria experts in setting district-level priorities and strategies for malaria
- Identify financing mobilization and management structures at community, district and national levels
- Identify effective strategies for cross-sectoral collaboration and strategic communications on malaria at the district level
- Identify effective approaches for mobilizing local authority figures (including traditional chiefs and medical practitioners) to support malaria elimination
- Assess the roles of women (and the impact of gender) and youth in local communities’ malaria elimination efforts

## Supporting information

S1 File Rethinking malaria: "Rethinking malaria in the context of COVID–19," a global engagement organized by Harvard University. (DOCX)
